# Assessment of implementation challenges of electronic medical record in Yekatit 12 hospital medical college

**DOI:** 10.1371/journal.pone.0329896

**Published:** 2025-08-06

**Authors:** Abiyot Alemu Mamae, Ephrem Mamo

**Affiliations:** 1 Department of Medicine, Yekatit 12 Hospital Medical College, Addis Ababa, Ethiopia; 2 Department of Public Health, Yekatit 12 Hospital Medical College, Addis Ababa, Ethiopia; Debre Berhan University, ETHIOPIA

## Abstract

**Background:**

The implementing challenges of Electronic Medical Records (EMR) refer to the difficulties and obstacles faced by healthcare organizations during the process of implementing and integrating electronic medical record systems into their operations. Research indicates that understanding the specific obstacles faced by healthcare organizations in implementing EMR is crucial for effective intervention strategies. Therefore, this study aims at assessing the implementation challenges of EMR.

**Methods:**

A cross sectional study design with quantitative and qualitative approach was employed in Yekatit 12 hospital medical college (Y12HMC). A structured and pre-tested questionnaire was used to analyse socio-demographics, knowledge, perceived benefits and usefulness, and challenges of EMR implementation. Descriptive statistics was employed to summarise the quantitative data. Qualitative data was thematically presented.

**Result:**

In this study 389 participants were surveyed with a response rate of 92.2%. Of the respondents, 54.4% had 1–5 service years, and 45% had no prior EMR system training. According to 89.7% of participants EMR improves practice productivity, and more secure than paper-based systems for storing patient records. Poor project management (62%), less eye contact with clients (57.6%), low users acceptance (55%), slow EMR system (45%), long waiting time for consultation (42.7%), lack of training and follow up (38%), high cost for wrong/repeated lab request (35.5%) and loss of privacy (33.2%) are challenges of implementation of EMR. The majority of the participants hold favorable perceptions and willing to use the system.

**Conclusion:**

The most common challenges for EMR implementation in Y12HMC are poor project management, less eye contact by physicians with clients, low users acceptance, lack of training and follow up and long waiting time for consultation. The majority of participants hold favorable perceptions and willing to use the system.

## Introduction

### Background

Health Information Technology (HIT) encompasses the utilization of computer hardware and software for managing healthcare information, data, and knowledge. Among HIT solutions, Electronic Medical Records (EMR) stand out as vital tools aimed at enhancing practice effectiveness and patient care [1]. Described by the World Health Organization (WHO) as digital versions of comprehensive patient data, EMRs offer various benefits such as reducing errors, enhancing clinical outcomes, and improving care coordination [2,3].

A comprehensive review of the existing literature underscores the multifaceted nature of EMR implementation challenges, revealing a complex interplay between technological constraints, organizational dynamics, and user perceptions. These findings underscore the complexity of the implementation process and highlight the need for tailored interventions to address specific barriers [4].

Numerous studies highlight challenges in EMR implementation, including resource shortages, technical issues, user resistance, inadequate training, data privacy concerns, system inoperability, insufficient management support and inadequate infrastructure [5–7]. Health professionals’ knowledge and perception of EMRs present additional hurdles to successful implementation [8].

While there is a growing consensus on the benefits of EMR adoption, divergent perspectives exist regarding the most effective strategies for overcoming implementation barriers. Some scholars advocate for top-down approaches, emphasizing the importance of strong leadership and organizational support, while others advocate for bottom-up strategies, highlighting the significance of frontline staff involvement and user-centered design principles [9–11].

Y12HMC is a pioneer government hospital in Ethiopia which has adopted EMR successfully across all services but faces gaps such as incomplete documentation, power interruptions, and difficulty accessing data, increased patient waiting time, difficulty of accessing laboratory results, and difficulty of getting monthly HMIS report. Healthcare providers’ perceptions of EMR also influenced its effectiveness. Failure to address these issues may lead to incomplete patient data, loss of information, decreased satisfaction, and poor care quality.

While physicians generally recognize the benefits of EMRs, face challenges such as slow typing processes, incomplete data input, lack of user-friendliness and difficulty accessing medical information, impacting their work efficiency and patient care [12]. A study reveals varying levels of knowledge and perception among healthcare providers regarding EMRs, highlighting the importance of assessing provider readiness and willingness to use the system [8].

Challenges specific to health institutes from workers perspective include data security risks, patient privacy concerns, and high infrastructure costs. Managerial support and effective change management are crucial for successful EMR implementation, emphasizing the need for clear stakeholder commitment and involvement throughout the process [13–15]. Challenges of EMR implementation also has challenges for patients which includes privacy concerns and reduced face-to-face communication during consultations [15].

Controversies also abound regarding the impact of EMR implementation on patient-provider interactions and clinical workflows. While proponents argue that EMRs streamline documentation processes and facilitate information sharing, critics raise concerns about the potential for technology-mediated communication barriers and increased administrative burden on healthcare providers [16,17].

There is limited research conducted addressing EMR implementation challenges after successful adoption. So the study aims to identify EMR implementation challenges in Y12HMC, potentially improving patient care and system efficiency.

## Method and material

### Study area

Yekatit 12 Hospital Medical College (Y12HMC) in Addis Ababa, Ethiopia, is the study site. Established in 1923 G.C. and initially named Bete Sayida (Teferi Mekonin) Hospital, Y12HMC became a medical college and research center in 2011 G.C. The hospital comprises 36 departments and 385 beds, offering a variety of preventive, curative, and rehabilitative health services to a population of about 4 million people. It employs more than 1,500 clinical, academic, and administrative support staff. Y12HMC has implemented Electronic Medical Records (EMR) across all service areas, except for ART clinics.

### Study design and period

A cross-sectional study design with both quantitative and qualitative approaches was conducted from February 2023 to June 2023, and participants were recruited between April 17, 2023 and May 1, 2023.

### Study population

The study population comprised clinical staff (specialists, general practitioners, nurses, laboratory scientists, radiology technologists, and pharmacists), hospital management staff (provosts, department heads, directors, and case team coordinators), and IT/HIT staff working during the study period.

### Inclusion criteria

Participants included clinical, management, and IT staff willing to give consent.

### Sample size and sampling procedure

A sample size of 422 was determined using stratified random sampling, comprising healthcare providers, IT staff, and administrative personnel, representing approximately 38% of the study population. The formula used was:


$$n=\frac{{\left(z\frac{\partial }{2}\right)}^{2}P(1-p)}{d^{2}}$$

z ∂/2 = 1.96(standard normal probability for 95% CI),p = proportion of the population which is 50% as there is no similar study done, q = 1-p and d = the degree of precision (for this case a 5% margin of error, i.e., d = 0.05). This calculation yielded 384, with a 10% non-response rate added, totaling 422. Stratified random sampling was employed to select participants, and purposive sampling was used for 10 in-depth interviews with key informants until data saturation.

Out of the 422 healthcare professionals selected for the study, 389 participants completed the survey, while the remaining participants did not return the questionnaire. This yielded a response rate of 92.2%. Stratified random sampling was employed to select participants from different groups (healthcare providers, IT staff, and administrative personnel) to ensure representativeness. In addition, purposive sampling was used for the in-depth interviews with 10 key informants, selected until data saturation was reached.

### Data collection procedure

Data were collected using a structured and pre-tested questionnaire that covered socio-demographics, knowledge, perceived benefits, usefulness, and challenges of EMR implementation. The questionnaire was pre-tested at Black Lion Hospital, a leading teaching hospital in Addis Ababa, Ethiopia, due to similarity in terms of system structure, staffing, and EMR exposure. This pilot test was conducted solely to evaluate the clarity, consistency, and reliability of the data collection tool. Data collection was supervised by an assigned supervisor and took a maximum of two weeks.

In-depth interviews were conducted with 10 key informants selected through purposive sampling. These individuals included healthcare providers, IT staff, and administrative personnel, and were chosen based on their expertise and direct involvement with EMR implementation. The interviews were semi-structured, allowing for flexibility in exploring participants’ perspectives on the topic.

Before each interview, participants were provided with an informed consent form explaining the purpose of the study, confidentiality measures, and their right to withdraw at any time. Interviews were conducted in person or via video conferencing, depending on the participant’s availability. Each interview lasted between 45 minutes and 1 hour, and the questions were open-ended to encourage detailed responses. Probing questions were used to clarify or further explore topics raised by the participants.

The interviews were audio-recorded with the participants’ consent, and detailed notes were taken during the session. Transcripts were generated from the recordings, ensuring that the data captured was accurate and comprehensive. Data saturation was achieved when no new information was emerging from the interviews, signaling that the core themes had been adequately explored.

### Variables of the study

**Independent Variables:** Age, sex, professional category, service year, management support, knowledge, perceived benefits and usefulness, infrastructure.
**Dependent Variables:** Challenges in EMR implementation (institutional and healthcare providers)

### Operational definitions

**EMR:** Digital medical records including patient health history, diagnoses, medications, tests, allergies, immunizations, and treatment plans.
**Implementation Challenges:** refers to difficulties encountered during the process of implementing and integrating EMR system into the health care practice. It includes issues related to human resources, staffing, infrastructure, resource allocation, leadership support, and processes.
**Perception of EMR:** is the attitudes, beliefs and feelings of healthcare providers and staffs towards the use and effectiveness of EMR systems in improving clinical practice and patient care.

### Data quality control measures

A structured chart was used to control data quality, measuring accuracy, relevancy, completeness, and timeliness.

### Data processing and analysis

Data were entered and cleaned using EPI-info 7.1.1 and exported to SPSS-V21 for analysis. Descriptive statistics summarized the data, with results presented in frequency tables and graphs. Qualitative data were thematically categorized and presented.

### Ethical considerations

Ethical clearance was obtained from Yekatit 12 Hospital Medical College. Permission to collect data was granted, and confidentiality was maintained throughout the study. Written informed consent was obtained from all participants before enrollment in the study, and all collected information was treated as strictly confidential.

## Results

### Sociodemographic characteristics of the study participants

Out of the 422 healthcare professionals chosen for the study, 389 participants answered to the survey, yielding a response rate of 92.2%. Sixty two percent of the participants were men. Among the study participants 61.7% of participants were between the ages of 22 and 31years. Fisty four percent of the participants have 1–5 years of service, while thirty six percent have between 6–10 years. General practitioners comprised 23.4% of the participants, specialists 18.3%, nurses 28.3%, lab technicians or scientists 11.6%, pharmacists 8.7%, and others (radiologists and anesthesiologists) 9.8%. Nurses represented the largest group among the respondents (Table 1).

**Table 1:** Sociodemographic characteristics of respondents, Y12HMC, 2023.

	Frequency	Percent (%)
Gender		
Male	241	62
Female	148	38
Total	389	100
Age range		
22-31	240	61.7
32-41	138	35.5
42-51	8	2.1
52-61	3	0.8
Total	389	100
Professional category		
Specialists	71	18.3
General practitioners	91	23.4
Nurses	110	28.3
Laboratory technologist	45	11.6
Pharmacists	34	8.7
Others	38	9.8
Total	389	100
Service years		
1-5	213	54.4
6-10	139	35.7
11-15	30	7.7
15 and above	7	1.8
Total	389	100

### EMR training and EMR knowledge

**Fig 1:**
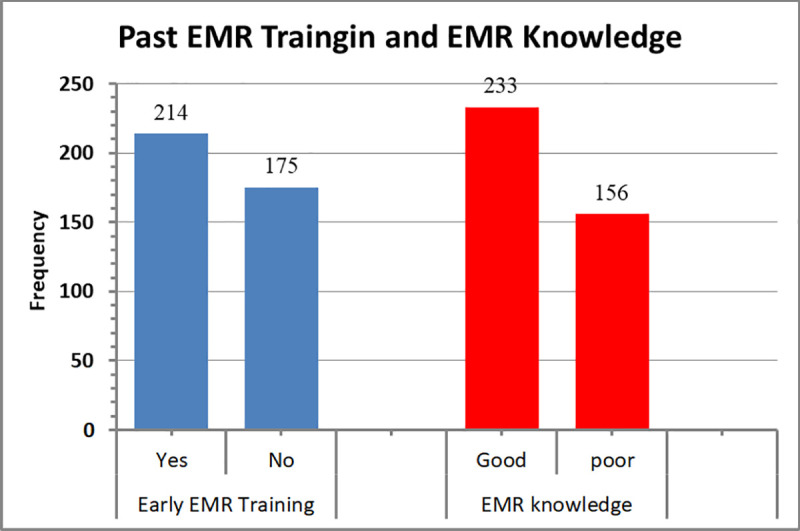
The Participant’s earlier EMR training and knowledge of EMR system, Y12HMC, 2023.

In response to a question on their prior system training attendance, 45% of respondents indicated that they had not gone to any prior EMR system training (Fig 1). In terms of their understanding of the EMR system, 60% of respondents have good knowledge about the system (Fig 1).

### Benefits of the EMR system

The majority of respondents (80.7%) thought that implementing the EMR system might significantly alter how patients are cared for. Additionally, 89.7% of those surveyed concurred that EMR had the advantage of boosting practice productivity (patients seen per day). According to 86.1% of those surveyed, using an EMR can help healthcare providers work less hard and more effectively and 94.1% of participants said that patient records available on EMR systems are safer and more secure than paper-based medical recording methods (Table 2).

**Table 2:** Views of respondents on the benefit of the EMR system, Y12HMC, 2023.

Items	Frequency	Percent (%)
EMR will change/already changed the process of patient care?		
Significantly	314	80.7
Small degree	57	14.7
Not at all	15	3.9
EMR will change/already changed the quality of care?		
Yes	354	91
No	35	9
EMR has a benefit of increasing practice productivity		
Yes	349.	89.7
No	40	10.3
EMR has benefit of decreasing the work load and enhancing efficiency of providers		
Yes	335	86.1
No	54	13.9
EMR system is safe and secured as compared to a paper based medical record system		
Yes	366	94.1
No	23	5.9

### EMR system contribution to service improvement

Three hundred fifty two (90.5%) of respondents stated that the EMR system lowered or enhanced service turnaround time based on their real day-to-day clinical practice (Table 3). Three hundred twenty two (82.8%) of respondents who evaluated the impact of EMR system downtime on clinical practices said it had a significant to serious impact on their clinical practice. Regarding the impact of the system on patient wait times, 38.8% saw a decrease in patient wait times, and 27.5% saw no change.

**Table 3:** Views on EMR system contributions to Service improvement, Y12HMC, 2023.

Items	Frequency	Percentage (%)
Services turnaround time improved/decreased by using EMR system		
Yes	352	90.5
No	37	9.5
System down time affects clinical practice?		
Very minimal effect	34	8.7
High effect	119	30.6
Serious effect	203	52.2
No effect at all	33	8.5
EMR system effect on patient waiting time?		
Reduces waiting time	151	38.8
Increased waiting time	131	33.7
No change	107	27.5

### Respondents perception and willingness to use EMR for future clinical practice

Overall, the analysis of the participating healthcare providers’ perceptions revealed positive insights. Results of the study on how health care providers perceived the system showed helpful perception, the easiness of the system (77.2%), their preference to use it (78.6%), completeness of patient information available on EMR (68.7%), care providers interest for involvement at the design and implementation phase (96.4%), and overall interest to use the system in future practice (82.8%) (Table 4).

**Table 4:** Respondents perception and willingness to use EMR for future clinical practice, Y12HMC, 2023.

Items	Frequency	Percentage (%)
It would be easy for me to become skillful at using EMRs		
Strongly disagree	22	5.7
Disagree	18	4.6
Don’t know	49	12.6
Agree	175	45
Strongly agree	125	32.1
I prefer EMR over paper charting		
Strongly disagree	19	4.9
Disagree	24	6.2
Don’t know	40	10.3
Agree	153	39.3
Strongly agree	153	39.3
Patient information obtained from EMR is more complete as compared to Paper medical record		
Strongly disagree	23	5.9
Disagree	39	10
Don’t know	60	15.4
Agree	171	44
Strongly agree	96	24.7
My involvement at the system design and during the EMR implementation phase will make the EMR more useful to me		
Strongly disagree	20	5.1
Disagree	18	4.6
Don’t know	54	13.9
Agree	194	49.9
Strongly agree	103	26.5
I would like to use EMRs in my work in future		
Strongly disagree	26	6.7
Disagree	17	4.4
Don’t know	24	6.2
Agree	165	42.4
Strongly agree	157	40.4

**Fig 2:**
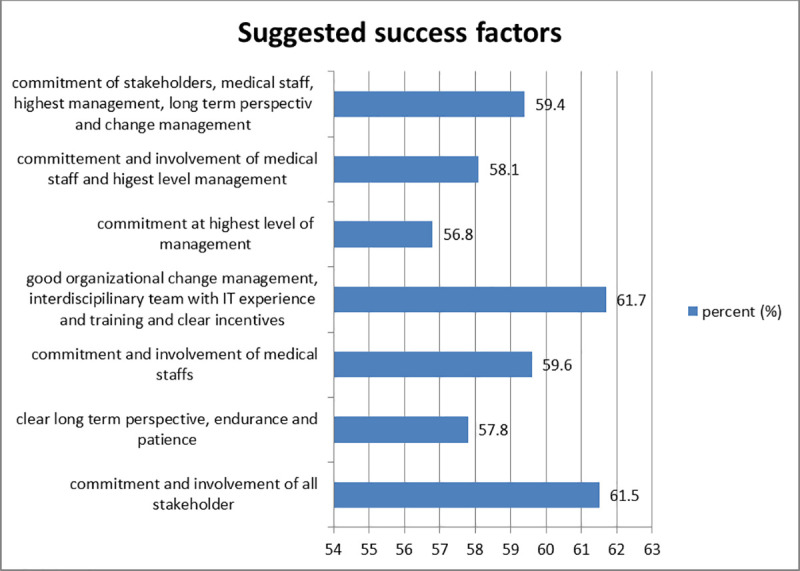
Suggested factors for successful implementation of EMR, Y12HMC, 2023.

The deployment of an EMR system had success elements identified by respondents. According to 61.7% of respondents, successful implementation would require the commitment and involvement of all medical professionals, good organizational change management, a multidisciplinary team with IT experience, training, and incentive systems (Fig 2).

### Result on implementation challenges for the hospital

**Fig 3:**
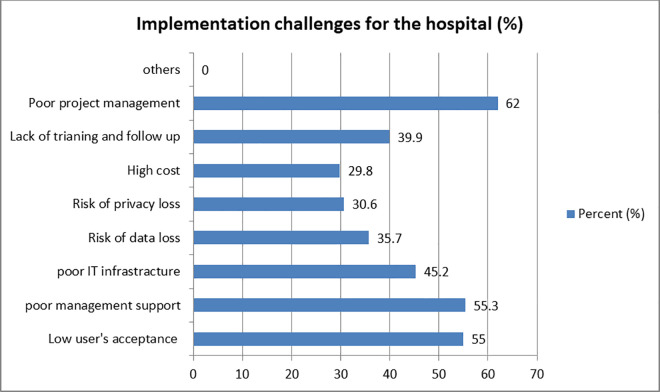
Implementation challenges for the Hospital, Y12HMC, 2023.

According to 35.7% of respondents, health institutions may be at risk of data loss, and 45.2% said the hospital’s IT infrastructure is inadequate. In circumstances of unauthorized information disclosure, 30.6% of respondents were concerned about the loss of patient privacy, particularly when administrative employees in addition to clinicians would have access to patient information in the EMR. Only 29.8% of respondents identified high costs as a challenge for the hospital in the system’s implementation. Poor project management (62%), inadequate management support (55.3%), low user acceptance (55%) and a lack of training and follow-up (39.9%) are among the main obstacles to the hospital’s adoption of the EMR (Fig 3).

**Fig 4:**
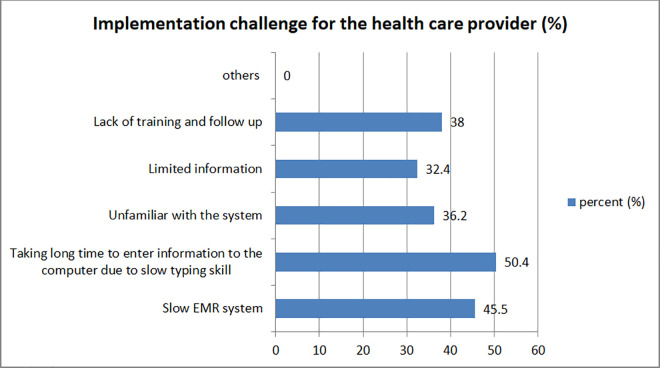
Implementation challenge for workers, Y12HMC, 2023.

The effectiveness of the system to streamline healthcare providers’ everyday tasks was a major concern since they were the primary users of EMR. The slowness of the system, slow typing speed of the provider, limited medical information retrieved from the EMR due to incomplete data input by other providers, lack of training and follow-up, and unfamiliarity with the system were the main obstacles for the providers to using the EMR, according to 45.5% of respondents, 50.4% of respondents, 32.4% of respondents, and 36.2% of respondents respectively (Fig 4).

### Results of implementation challenges for patients

**Fig 5:**
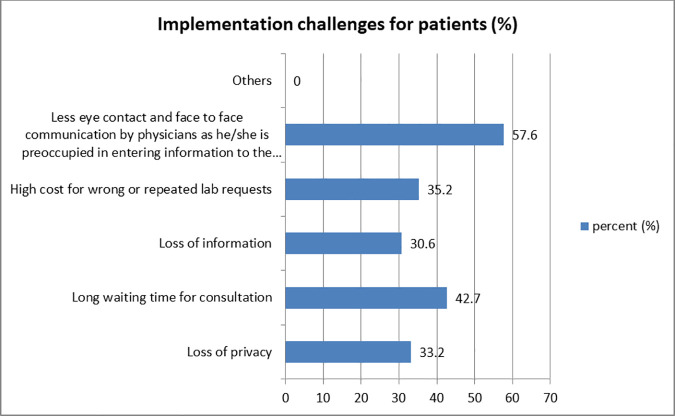
Implementation challenges for patients, Y12HMC, 2023.

Despite the fact that there are currently privacy protections in the EMR, 33.2% of respondents are concerned about patient privacy. According to 57.6% of respondents, face-to-face interaction and eye contact can be less frequent when providers are focused on entering data into a computer during a consultation. Thirty one percent and 42.7% of respondents suggested loss of information and long waiting time for consultation respectively as a challenge of implementation of EMR for patients (Fig 5).

### Results from the qualitative data

The findings from the qualitative data explored overall views and detailed stories from the hospitals’ management, EMR system administrators, and IT support personnel. These results were believed to reinforce the quantitative results and were therefore presented below. Additionally, a summary of general themes, sub-themes, and categories were presented (Table 5).

**Table 5:** Summary of qualitative data general themes, sub-themes and response categories, Y12HMC, 2023.

Theme	Sub-theme	Response categories
Implementation of EMR	Deployment goals The system’s condition at the moment	Improved efficiency and service quality while lowering healthcare costs. High dependency on the system. Performing well, third phase on progress
ICT infrastructure	IT facilities and equipments	Network infrastructure Availability of equipment and facilities
Organization	Management commitment	Well planned Providers involvement Training and follow up Top level management commitment
Implementation challenges	Providers	Resistance from care providers Lack of computer typing skill Lack of training Slow system Low staff awareness
	Organization	Poor project management Low users acceptance Risk of privacy loss Risk of data loss
	IT related	Shortage of equipments Slowness of the system Data vulnerability Lack of technical expertise

#### EMR system implementation objectives, deployment efforts and current status.

The study’s findings demonstrated that the hospital’s management had planned to switch from paper-based record keeping to an electronic recording system by “implementing the EMR system, which was determined to improve the quality and efficiency and, above all, to reduce hospital costs” (Higher Hospital Management 001, 002, 006). Interviewees stated that “this aim was accomplished at Y12HMC, and the system is still in good working order. All medical services are currently dependent on the system. In collaboration with the representative from each department, we formed an EMR committee, and as a result, we were able to provide uninterrupted IT assistance for 24 hours (Higher Hospital Management 001, 002).

The EMR project in Y12HMC was started in 2020; however it was implemented in three phases. “The initial phase started by establishing reception, triaging, and billing windows,” the respondent said. Then outpatient, inpatient, pharmacy and diagnostics followed in the second phase which required the bulk of data encoding takes place from the paper based recording system to the EMR system. Currently the third and final phase of dashboard, reporting and integration phase is on progress (Higher Hospital Management 001, 002, Sys Admin 004). “All service delivery points currently use EMR, although several features—like the certificate and referral out functionalities—don’t operate. Some departments have begun using system registers and reports for dashboards, while others still rely on manual reporting methods.” (Sys Admin 004)

#### ICT infrastructures and availability of technical support staff.

The outcome showed that the hospital’s ICT infrastructure was in good shape. “During the deployment of the EMR system, staffs from Pulse-tech in collaboration with staffs from the Addis Ababa health bureau and trained physicians from the hospital were responsible for providing all technical support,” the respondent retorted. These included setting up the program and configuring it, providing the necessary hardware, performing system backups, educating the IT personnel and caretakers, and monitoring the project (Sys Admin 003).

The respondent went on to say that “later, the development of an ICT infrastructure throughout the hospital with IT facilities, equipment, and a well-equipped data center, fortunately, supported the implementation of the EMR and contributed to having a sustainable system to the present day.” (“Sys Admin 003”). The wired network connection was another significant issue raised by respondents. “The network is primarily based on the wired network connection which is more secure and not prone to interference by other networks” said one interviewee. Additionally, it is not slow compared to wireless network connections (Sys Admin 003, Sys Admin 004).

One of the respondent said that “departments have adequate computer except few departments where the capacity of the computer is not appropriate and few departments also reported computer shortage “(“Sys Admin 004”).

### Implementation challenges of EMR

System administrators’ findings indicate that implementation obstacles differed depending on the phase of deployment, stating that “at the first phase, when our focus was on coverage (to fully integrate EMR), the biggest challenge was professionals’ opposition, unfavorable attitudes, and infrastructure and hardware shortage. After full-scale EMR implementation, the main challenge is improper professional use of the system (as the system is designed) – they fill out inappropriate data, low tolerance level for minor system problems, lack of awareness about the system, inadequate implementation support by hospital staffs, Not having adequate IT and EMR support staffs, underestimation of the use and overall impact of EMR on health service delivery. Lack of a structure to provide orientation to interns and residents was the fundamental issue in all phases, and we have no mechanism to measure their performance at the end”. Lack of standard work flow and standard format for some departments affect both the implementation and maturity of the system.(“Sys Admin 004, 006, 003”).

Findings from the senior management and IT experts’ side quoted the response as follows: “The challenges include interruption of service when electricity is out, low staff awareness, low level of commitment from the midlevel management, lack of integration and interoperability with other systems like laboratory and pharmacy, system owner delays in fixing problems,” (“Sys Admin 004, IT personnel 002)

A participant in the interview stated that one of the challenges in implementing an EMR was low staff awareness: “Only a few interested staffs know the details of the system and use the system properly; while the majority of providers don’t have adequate knowledge of the system even the specific module they have used.” (“Sys Admin 004”).

### Success factors

Participants in the interview indicated a number of factors for the EMR system to be successful in their hospital. The response from the interviewees quoted as follows; “The sustainability of the EMR system depends on the existence of strong EMR support team with the required professional mix, leadership support, availability of sufficient ICT infrastructure with accessories, establishment of daily audit and feedback system, maintenance capacity of the vendor, the architecture and design of the system-able to manage complex modules and large number of users and any system associated problems solved easily. Concern, willingness, and involvement of medical staffs and initiation of duty system as incentives for IT support staffs contributed a lot” (higher hospital management 001, Sys Admin 004, IT personnel 003).

## Discussion

An EMR system that is acknowledged for supporting a high-quality, integrated health care information system that is independent of the place and time of health care delivery through information communication technology [18] is perceived to increase efficacy and boost efficiency of health care delivery [13]. Y12HMC implemented an EMR system to improve healthcare delivery efficiency and efficacy. The system, developed with Addis Ababa health bureau and ICT agency, aimed to support interoperability, reduce medical errors, increase legibility of medical records, and decrease hospital costs. The system was developed in three phases, starting with reception, triage, and billing, and then outpatient, inpatient, pharmacy, laboratory, and imaging services. The EMR is now operational with periodic system improvements.

According to the analysis from this research, 55.0% of respondents had taken advantage of the institution’s earlier EMR system training. This result, 55.0%, is better compared to the result of previous study on different study area in Ethiopia (only 37.0% got training) [19] and it is low compared to other study (64.0%) [8] Literatures suggested before any extensive EMR adoption, training and follow-up as crucial components of actualization [6,20]. One study’s finding highlighted the significance of learning at least the fundamentals of how to use the software system before implementing an EMR system [20].

The relationship between training and positive perceptions of the EMR system is clear. Adequate training is linked with higher user confidence and better system acceptance. The findings suggest that while there has been progress in training (55%), this remains insufficient to ensure widespread proficiency. Hospital management should enhance training programs by introducing more comprehensive and ongoing training sessions to ensure that all staff, from junior to senior professionals, is equipped with the skills necessary for effective system utilization. This will improve user adoption and satisfaction in the long term.

In this research 90.5% of respondents stated that the EMR system lowered or enhanced service turnaround time and 82.8% said EMR system downtime has a significant to serious impact on their clinical practice. Regarding the impact of the system on patient wait times, 33.7% of respondents saw a rise in patient wait times, 38.8% saw a decrease in patient wait times, and 27.5% saw no change. These findings are supported with a research at a referral hospital in Kigali, Rwanda where 88.2% of participants thought the standard of service had increased since the implementation of the Open Clinic EMR. These findings are supported with a research at a referral hospital in Kigali, Rwanda where 88.2% of participants thought the standard of service had increased since the implementation of the Open Clinic EMR [18]. In similarly vein, 80.6% of respondents believed that the system had significantly improved patient care, reducing waiting times and improving record accuracy and safety [18].

While service turnaround times improved for many, the variation in patient wait times reflects that EMR implementation is not a one-size-fits-all solution. Some areas of healthcare delivery benefited more than others, and workflow integration issues may explain the discrepancies observed. To optimize EMR performance, hospitals should conduct workflow analyses to identify specific bottlenecks caused by system use. These findings highlight the importance of customized solutions that consider hospital-specific needs and the role of the EMR in enhancing patient flow.

In this research 60% of respondents have good knowledge about the EMR system. This is higher than a study in other area of Ethiopia, where only 54.5% had good knowledge about EMR. The health professionals who have good knowledge have the tendency to accept the advantage of EMR system [8]. The majority of participants, 77%, hold favorable perceptions and willing to use the system in this study, which is in alignment with other research [5].

The link between knowledge and perception suggests that well-informed healthcare professionals are more likely to embrace the EMR system. The study highlights the role of comprehensive training and experience in shaping healthcare providers’ views of the system. To improve user acceptance and overall system utilization, healthcare organizations should invest in education initiatives tailored to different knowledge levels. Increasing staff awareness and understanding of EMR functionality will enhance their confidence in its use and contribute to greater operational success.

In this study the implementation challenges of EMR are assessed from health care providers perspective in three categories; challenges for the institute, for health care provider and for the patients. The following was listed by study participants as the primary challenge to EMR use for health care providers. These include system’s slowness (45.5%), slow typing speed of providers (50.4%), limited medical information retrieved from the EMR due to incomplete data input by other provider (32.4%), lack of training and follow up (38%) and unfamiliarity with the system (36.2%). For 91% of the responders, the quality of care had improved. The use of the EMR had also raised practice productivity, reduced workload, and improved the effectiveness of healthcare professionals. In addition 94.1% of the respondents agreed that patient records stored on an EMR system are more secure and safe than those kept on paper-based medical record systems. These findings are in agreement with the findings of researches [21–23].

While EMR systems were seen to improve quality of care, operational challenges such as system slowness and data integrity issues could undermine these benefits if not addressed. The system’s security benefits were noted positively, indicating its potential to improve patient confidentiality. To fully capitalize on the security benefits of EMR, healthcare organizations should focus on enhancing system speed and ensuring accurate data entry. Additionally, hospitals must prioritize follow-up training to ensure that users can leverage the system’s capabilities effectively, without technical obstacles slowing down performance.

From the health care perspectives, the major challenges for patients in the implementation of EMR are loss of patient privacy (33.2%), less face-to-face interaction and less eye contact between health care provider and the patients (57.6%), loss of information (30.6%), unnecessary costs due to wrong tests (35.2%), and long waiting time for consultation (42.7%). These findings are very high compared to one research where only 12.5% of health care providers are concerned for loss of patient privacy, 6.3% suggested loss of eye contact and it says EMR reduces duplicated medical tests [15,24]. A study in Kenya suggested patients spent less time waiting to see a consultant (24%) [20].

The increased concern over privacy loss and diminished personal interaction suggests that patients may feel alienated by the technological shift, highlighting the need for balance between technology and human care. Hospitals must ensure that the privacy of patient records is prioritized, with strong security protocols in place. Additionally, to address concerns about personal interaction, healthcare facilities should incorporate strategies to maintain patient-provider rapport during consultations, even in the age of digital health systems.

Several studies have pointed to common barriers in EMR implementation, such as a lack of human resources, hardware and software malfunctions, user resistance, lack of technical expertise, insufficient ICT infrastructure, and lack of awareness [6,13,14,25,26]. This study found similar issues, including poor project management, lack of training, poor user acceptance, and slow system performance. These barriers align with the literature, which emphasizes that the implementation of EMR system is often hindered by cost, privacy concerns, resistance, and lack of technical support.

The study confirms that implementation challenges are multifaceted, involving not only technical issues but also cultural and organizational resistance. These barriers need to be tackled through a holistic approach that addresses both technology and human factors. Healthcare leaders must take a multidimensional approach to tackle barriers. Cross-departmental collaboration, strong project management, and continuous staff engagement in the process are essential for overcoming these obstacles and achieving successful EMR implementation.

The responses from system administrators offer valuable insights into the various challenges encountered the different phases of EMR deployment. This illuminates the complex landscape of EMR implementation and presents a multifaceted picture of the obstacles faced by healthcare organizations.

The administrators observed a shift in challenges during EMR deployment. During the initial phase, the focus on achieving comprehensive integration of EMR into the healthcare environment brought forth significant hurdles. These include resistance, negative attitudes, and infrastructure constraints. Transitioning to full-scale implementation revealed issues like incorrect data entry, low tolerance for setbacks, lack of awareness, inadequate support, and insufficient IT resources. These results are consistent with those found in the literatures [5,6,27].

The challenges faced by administrators underscore the importance of addressing both technical issues and organizational dynamics during the deployment phase. Ongoing technical support and change management strategies are crucial for long-term success. Healthcare organizations should not only invest in system upgrades but also develop comprehensive post-implementation strategies, including ongoing user training and support, to ensure continued system success and minimize operational challenges.

The interview finding highlights the need for structured orientation programs for interns and residents, comprehensive onboarding strategies, and performance evaluation mechanisms. It emphasizes the importance of organizational culture and management in successful EMR implementation, highlighting the need for systemic changes in education, training, and performance assessment protocols. Studies also support this findings [14,20].

The responses from senior management, IT experts, and interview participants shed light on several critical challenges affecting the successful implementation of EMR systems within the healthcare environment. An important factor highlighted was the vulnerability of the system to service interruptions during power outages, underlining the significance of robust infrastructure and contingency plans to maintain system operability (Sys Admin 004, IT personnel 006). Moreover, the issue of low staff awareness emerged as a significant barrier, with a participant highlighting the divide between a minority of well-informed, engaged staff members and a majority with inadequate knowledge about the EMR system and its specific modules (Sys Admin 004). These findings are similar to the findings of the quantitative part of this study and are also supported by literatures [5,6].

The interviewees highlighted key factors for successful EMR system deployment in their hospital environment, including a strong support team, leadership, robust ICT infrastructure, regular audits, vendor maintenance, efficient system design, and efficient resolution of system-related issues. They also highlighted the importance of medical staff involvement and incentive structures for IT support staff, emphasizing the multidimensional nature of EMR implementation. The results of literature provide support for these findings [13,14].

## Conclusions

The study examines the challenges of EMR implementation from the perspectives of healthcare providers for the three stakeholders. While EMR systems have improved care quality, practice productivity, and data security, they also present challenges such as system inefficiencies, patient privacy concerns, and prolonged waiting times. The study highlights the importance of structured orientation programs, robust infrastructure, and knowledgeable staff in overcoming these obstacles. The study highlights the complex interplay of technical, human, and systemic factors in EMR deployment.
